# A novel protein ubiquitination-related five-gene signature predicts overall survival in patients with lung adenocarcinoma

**DOI:** 10.18632/aging.202663

**Published:** 2021-03-10

**Authors:** Ran Xu, Tong Lu, Jun Wang, LinYou Zhang

**Affiliations:** 1 Department of Thoracic Surgery, The Second Affiliated Hospital of Harbin Medical University, Harbin, China; 2 Harbin Medical University, Harbin, China

**Keywords:** ubiquitination, lung cancer, adenocarcinoma, signature, prognosis

## Abstract

Protein ubiquitination has been reported to be involved in many biological processes that affect cancer cell growth or death. In this study, we identified differentially expressed E3s/DUB-related genes associated with the prognosis of lung adenocarcinoma and then constructed an E3s/DUB enzyme signature prediction model for the training group and validated its accuracy for prognosis prediction in the validation group. According to our constructed model, all patients were divided into the high- or low-risk group, and a comparison of the two groups revealed that the high-risk group had poorer survival and higher mortality than the low-risk group. The calculated risk score was also an independent prognostic factor when analyzed together with other clinical factors. To explore the functions of the signature genes, we predicted the substrate proteins with which they interact and then performed enrichment analysis. Interestingly, we found that the signature genes were enriched in multiple treatment resistance and immune-related pathways. Therefore, we continued to analyze immune infiltration in the samples and found a variety of differences in immune cell infiltration. According to our constructed model, these differences in immune cell infiltration may predict different immune statuses after grouping and are associated with worse prognosis in high-risk patients.

## INTRODUCTION

Lung cancer is the deadliest malignancy worldwide [1], and its 5-year survival rate is approximately 4-17% [2]. The pathological types of lung cancer include non-small cell lung cancer (NSCLC) and small cell lung cancer (SCLC). NSCLC is further divided into adenocarcinoma (LUAD) and squamous cell carcinoma (LUSC), of which adenocarcinoma is the most common type. For last decades, it was believed that smoking was the primary cause of NSCLC. However, the incidence of NSCLC in non-smokers is increasing year by year [3], but the specific reason is still unclear. Surgery is considered to be the gold standard in treating early-stage lung cancer [4], while radiotherapy, chemotherapy, targeted therapy, and immunotherapy are also available for patients with advanced disease [5]. In recent years, EGFR-TKIs targeting EGFR mutations have shown significant effects [6]. The application of the immune checkpoint inhibitors PD-1 and PD-L1 also provides a broader strategy for the comprehensive treatment of lung adenocarcinoma [7]. Thus, exploration of novel approaches for monitoring prognosis and improving LUAD immunotherapy is urgently needed.

Ubiquitination is a type of post-translational modification that participates in regulating protein function or degradation by the proteasome [8]. The process of protein ubiquitination involves three types of enzymes: ubiquitin-activating enzyme (E1), ubiquitin-coupled enzyme (E2), and ubiquitin ligase (E3) [9]. The E1 enzyme is responsible for the adenylation of ubiquitin, a process that consumes one molecule ATP, and the E2 enzyme transports ubiquitin that is adenylated by E1 to E3 [10]. Finally, different E3 enzymes recognize diverse substrate proteins and catalyze ubiquitin transfer from E2 to the lysine (K) residues of substrate proteins to complete monoubiquitination or polyubiquitination of substrate proteins [11, 12]. Indeed, like other posttranslational modifications, ubiquitin chains on the lysine (K) residues of substrate proteins can be removed by deubiquitinases (DUBs, also known as deubiquitinating enzymes). The most classical function of ubiquitination and deubiquitination is to mediate the degradation of a protein by the 26S proteasome or stabilize it [13]. However, it has been reported that ubiquitin enzymes (UBs) and deubiquitinases (DUBs) are associated with various biological processes, such as the cell cycle, transcription, signal transduction, apoptosis, the immune response, protein interactions, and subcellular localization [14–16]. Among these enzymes, E3s and DUBs have been shown to bind specifically to substrates [17].

In lung cancer, several studies have reported that E3s/DUBs can interact with their substrates and play significant roles in LUAD. For example, it has been demonstrated that USP7 Monoubiquitination Histone H2b sensitizes lung cancer cells to ferroptosis [18]. Furthermore, UBE2O mediates Mxi1 ubiquitination and degradation to promotes lung cancer cell proliferation and radioresistance [19]. An E3 ubiquitin ligase complex CRL3(BTBD9) targets TNFAIP1 for degradation to suppress lung cancer cell migration. Moreover, the mRNA expression of BTBD9 is associated with the overall survival in lung cancer patients [20]. Thus, it is believed that constructing an E3s/DUB-related gene prediction model to monitor LUAD patients' prognosis would be valuable and that timely intervention can significantly improve the prognosis of patients with high-risk scores. In this study, we analyzed E3s/DUB-related gene expression associated with patients’ clinical outcomes obtained from The Cancer Genome Atlas (TCGA) database. A prognostic prediction model for LUAD patients was constructed. The substrate proteins of these signature genes were next predicted. Besides, functional enrichment analysis of the substrate proteins showed that they might affect sensitivity to chemotherapy, targeted therapy, and immunotherapy.

## RESULTS

### Grouping of samples and identification of prognostic E3s/DUB-related DEGs

**Figure 1:**
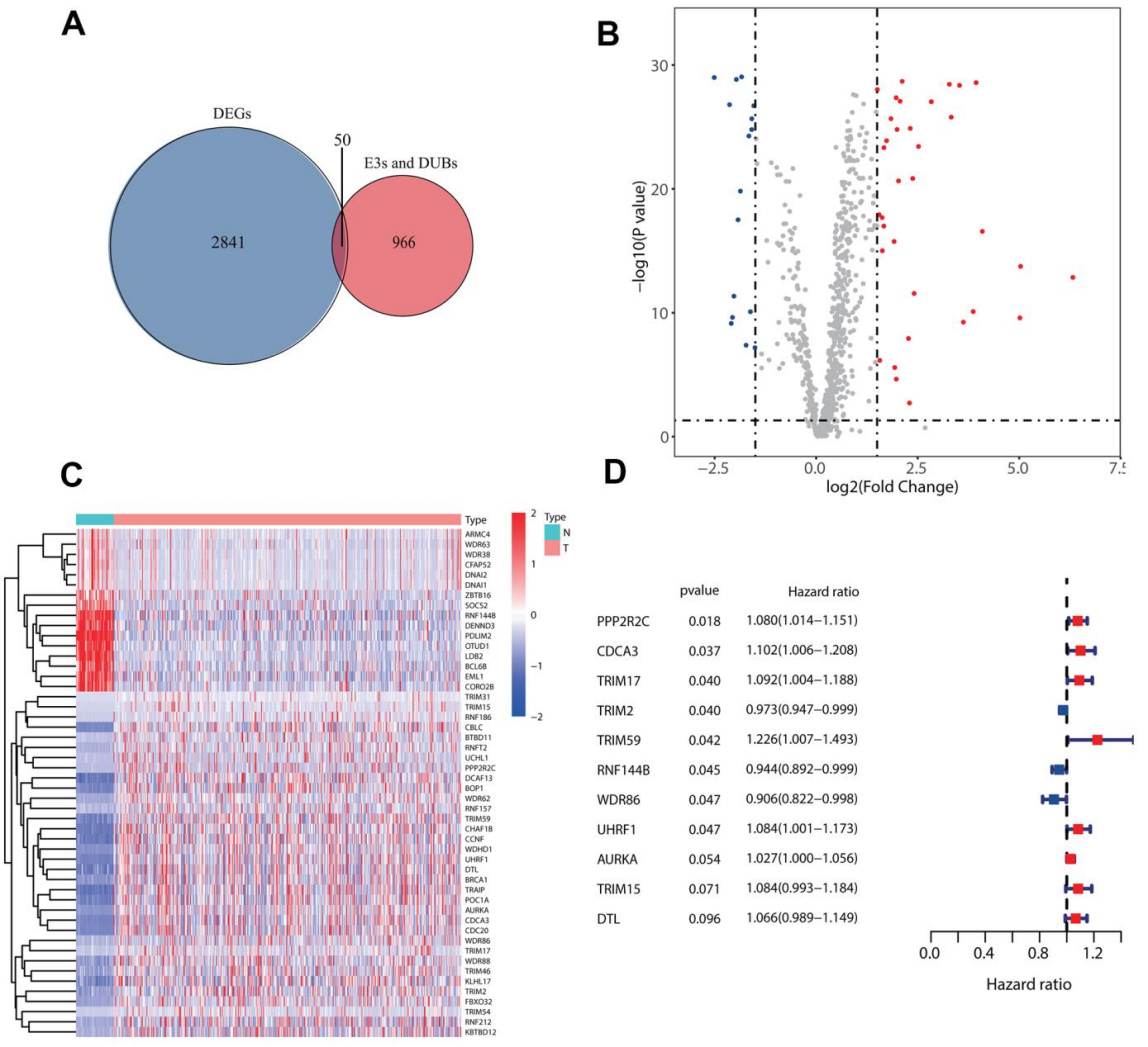
**Identification of prognostic E3s/DUBs related DEGs.** (**A**) Veen Diagram for the differentially expressed genes (DEGs)of all samples' whole gene expression with all E3s/DUBs-related genes. (**B**) Of the 50 E3s/DUBs-related DEGs, 34 were up-regulated, and 16 were downregulated. (**C**) Heatmap of E3s/DUBs-related DEGs in all samples. (**D**) Univariate Cox regression analysis showed that 11 genes were associated with overall survival (*P*<0.1).

First, 50 E3s/DUB-related genes were identified to be differentially expressed (Figure 1A), including 16 that were downregulated and 34 that were upregulated (Figure 1B). Besides, a heatmap was generated to show the expression levels (Figure 1C). Next, we randomly divided the samples with complete survival information into the training group (n=227; 50%) and test group (n=227; 50%). A total of 11 E3s/DUB-related DEGs were identified to be associated with overall survival according to the results of a univariate Cox regression analysis of the training group, with eight genes (PPP2R2C, CDCA3, TRIM59, UHRF1, AURKA, TRIM15, DTL) predicting poor outcome with a hazard ratio (HR) over 1 and three genes (TRIM2, RNF144B, WDR86) predicting good outcome with a hazard ratio (HR) less than 1. (Figure 1D).

### Construction of a prediction model with the five E3s/DUB-related signature genes

**Figure 2:**
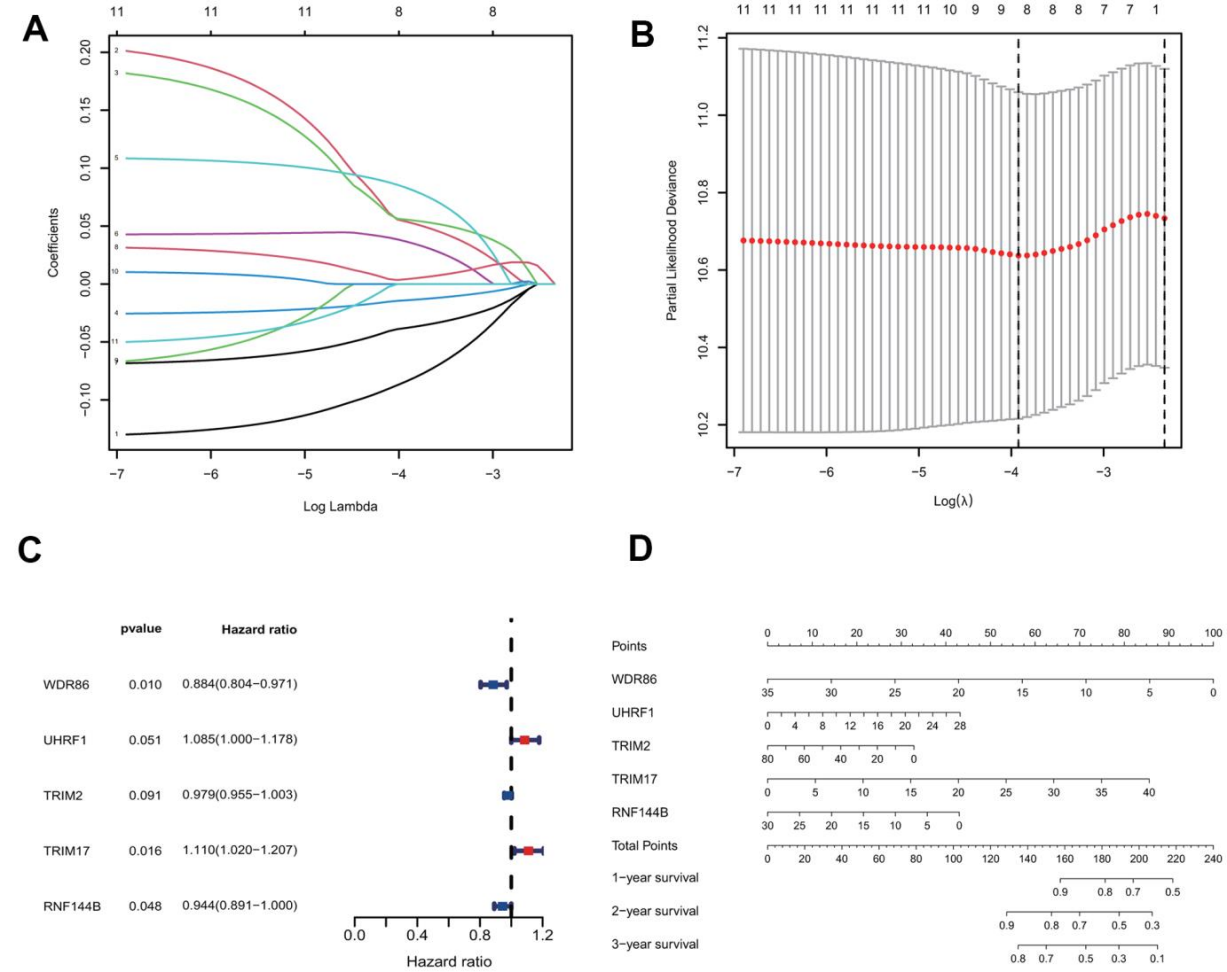
**Construction of the predictive five E3/DUB-related genes signature model in the training group.** (**A**) LASSO coefficient profiles of the expression of 11 candidate genes. (**B**) Confidence intervals for each lambda. (**C**) Multivariate Cox analysis of 8 genes derived from the Lasso. (**D**) A nomogram of the five-gene model predicting patient outcome.

To construct a prognostic model for predicting survival, we fit the 11 genes mentioned above to the LASSO-Cox regression model. Eight robustly expressed E3s/DUB-related prognostic genes (WDR86, UHRF1, TRIM59, TRIM2, TRIM17, TRIM15, RNF144B, PPP2R2C) performed well in the LASSO analyses of the training group (Figure 2A, 2B). Based on the LASSO results, eight robustly expressed genes were used to perform multivariate Cox regression analysis. Eventually, five genes were selected to construct the risk score model (Figure 2C). The risk score of each patient was calculated as following according to the coefficients: risk score= (-0.12371* WDR86)+(0.081659* UHRF1)+(-0.02155* TRIM2)+(0.103937* TRIM17)+(-0.05769* RNF144B). Additionally, a nomogram of the predictive model for patient survival based on five genes was plotted (Figure 2D).

### Evaluation of the predictive ability of the five signature genes for overall survival in the training group and test group

**Figure 3:**
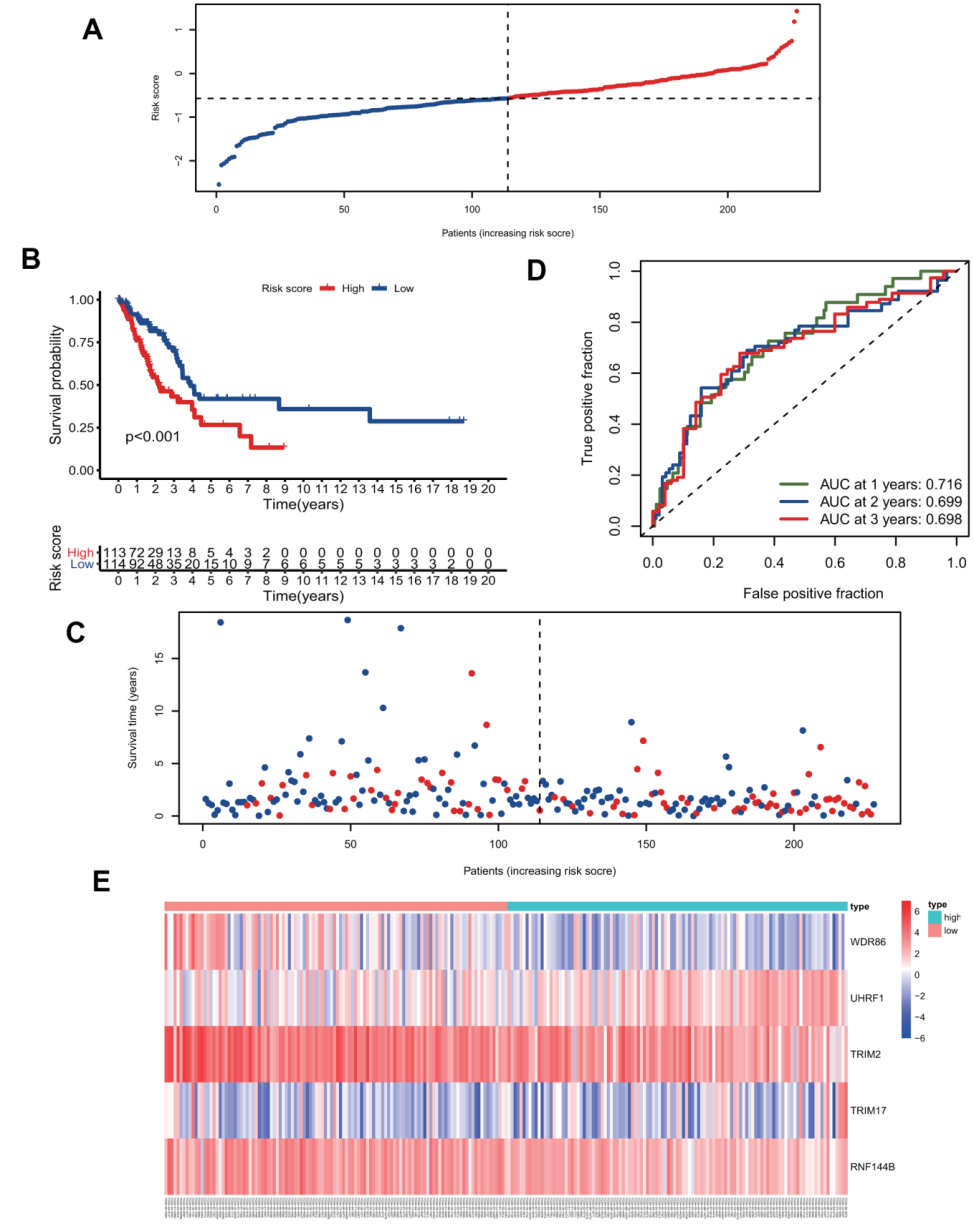
**The five-gene signature predicts overall survival in the training group.** (**A**) The distribution of risk-score and patients' grouping. (**B**) Kaplan-Meier survival curves of high and low-risk patients. (**C**) Vital status of patients. (**D**) ROC curves of the predictive model in the training group. (**E**) Heatmap of five genes in the training group.

**Figure 4:**
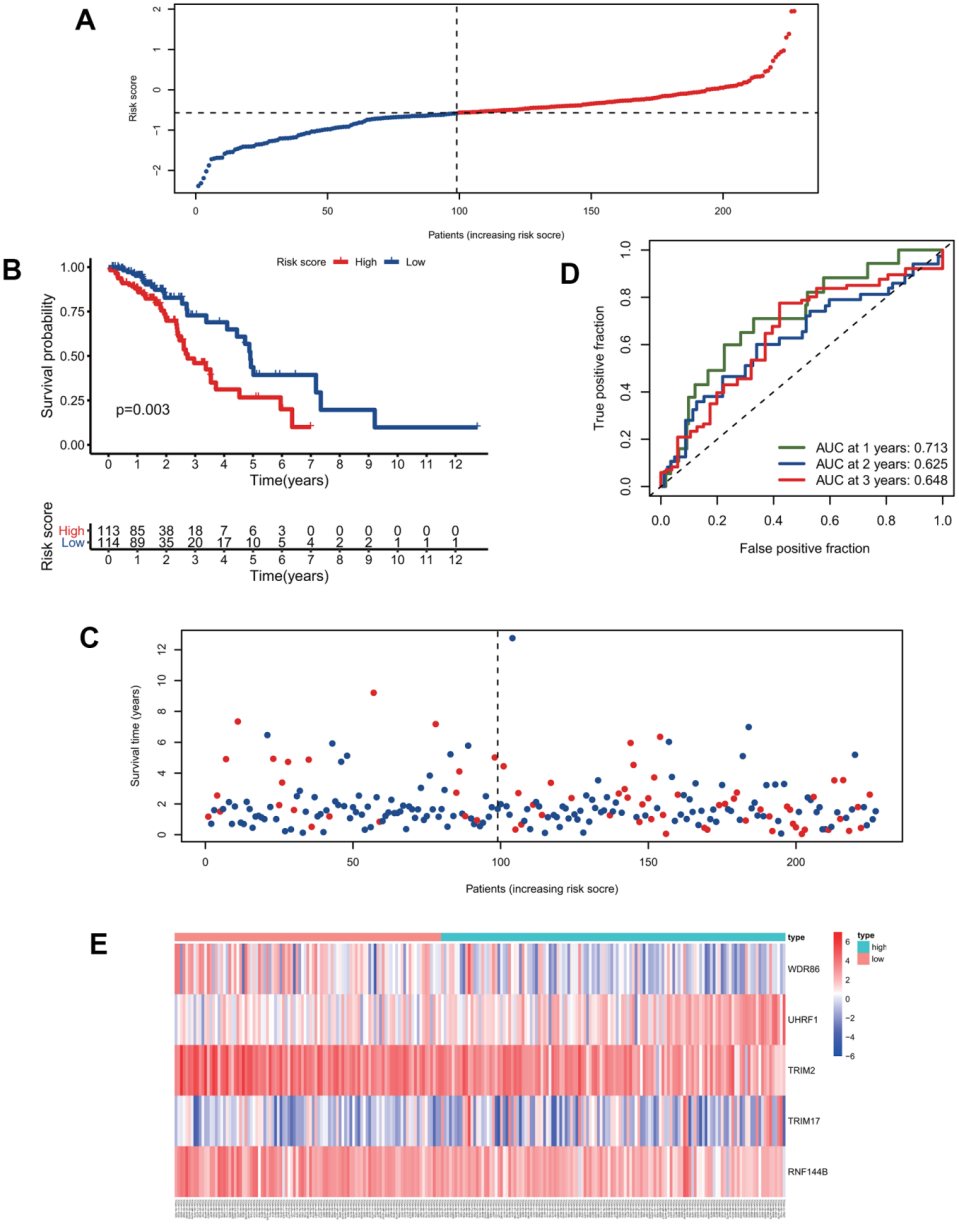
**Validation of the predictive model.** (**A**) The distribution of risk-score in the test group. (**B**) Kaplan-Meier survival curves of high and low-risk patients in the test group. (**C**) Vital status of patients in the test group. (**D**) ROC curves of the predictive model in the test group. (**E**) Heatmap of five genes in the test group.

We calculated the risk scores of all patients in the training group using the five signature gene prediction model and classified patients into high-risk and low-risk groups according to the median risk score values (Figure 3A). Kaplan-Meier log-rank analyses showed that patients with high risk had a worse survival than patients with low risk in the training group (Figure 3B). Moreover, the mortality rate of patients in the high-risk group was higher than that of patients in the low-risk group (Figure 3C). The prediction model's ROC curves at 1, 2, and 3 years all showed that the model accurately predicted patient survival (Figure 3D). Additionally, the heatmap showed the landscape of the five signature genes in the training group (Figure 3E). To verify the model's reliability, we calculated the risk score of all patients in the test group and then used the same cutoff value as that used for the training group to group the patients (Figure 4A). As shown in Figure 4B, 4C, in the test group, high-risk patients had a significantly worse prognosis and mortality rate than low-risk patients. Besides, the ROC curve for the test group proved that the model was reliable (Figure 4D). Additionally, we visualized the expression of five signature genes in the test group (Figure 4E).

### Risk score is a better independent prognostic-related factor than other clinical factors

**Figure 5:**
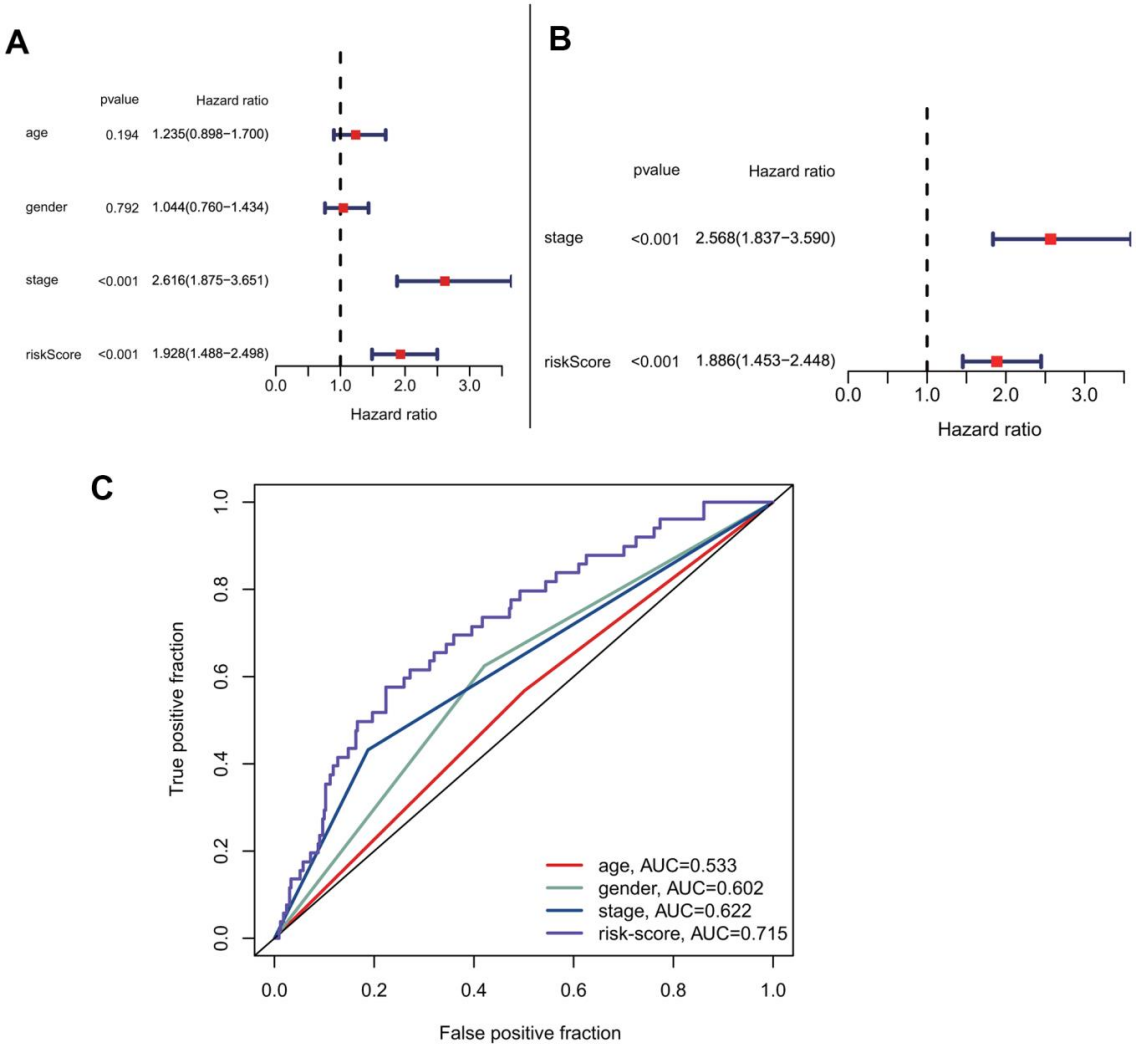
**Prognostic correlation analysis with risk-score and other clinical characteristics.** (**A**) Univariate analysis of risk-score and other clinical features. (**B**) Multivariate cox analysis showed that risk-score was an independent prognostic factor. (**C**) Comparison of the accuracy of risk-score and other clinical characteristics in predicting patients prognosis.

To explore the predictive model's relationship with other clinical factors, we systematically profiled the risk scores and clinical characteristics of all patients, such as age, gender, and stage (Supplementary Table 1). Univariate Cox regression analysis showed that risk score was more strongly associated with overall survival than other factors (Figure 5A). Additionally, multivariate Cox regression analysis showed that risk score was a better independent prognostic factor than other clinical factors (Figure 5B). The ROC curves for risk score and other clinical characteristics also showed that risk score had a better predictive ability than other clinical factors (Figure 5C). To compare the predictive effect of our constructed model on prognosis with other clinical factors, we further constructed decision curves for risk-scores that calculated by our prognostic model and other clinical factors (Supplementary Figure 1A, 1B). The results showed that the risk-scores calculated by our prognostic model had better benefits than other indicators as clinical intervention indicators.

### Prediction of the substrate proteins of the signature genes and their functional analysis

**Figure 6:**
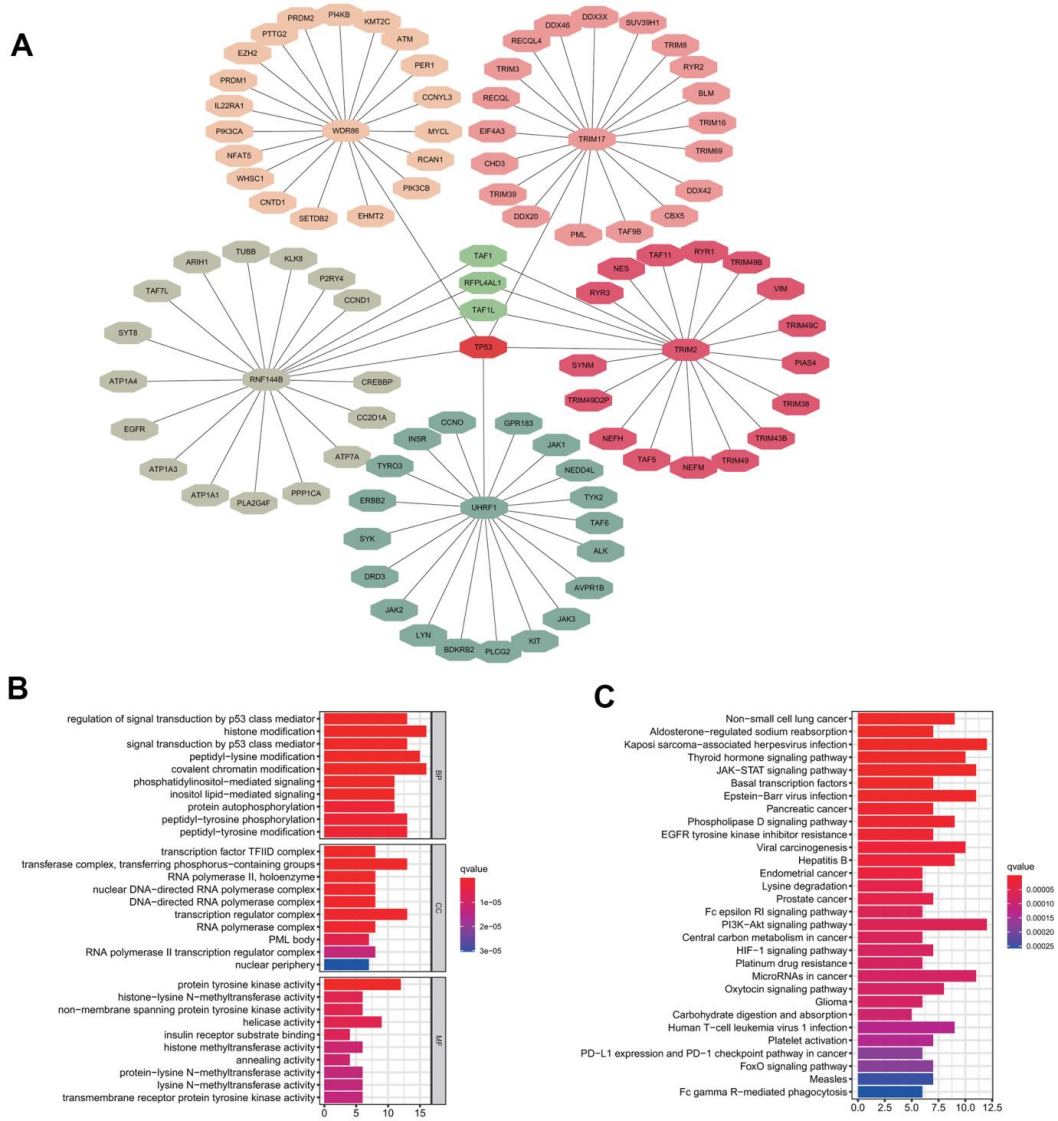
**Substrate protein prediction and potential functional analysis.** (**A**) The prediction of substrate proteins. (**B**) GO enrichment analysis. (**C**) KEGG pathway enrichment analysis.

To investigate the potential functional impact of the five signature genes, we predicted the likely substrate proteins of the signature genes using “UbiBrowser”. The top 20 substrate proteins of each signature gene, which were identified according to their predicted scores were selected for further functional analysis (Figure 6A). GO enrichment analysis showed that all substrate proteins were involved in regulating the classical P53 pathway and various epigenetic regulation mechanisms, including histone modification, chromatin modification, and protein modification (Figure 6B). Additionally, KEGG analysis suggested that the substrate proteins were mainly enriched in the non-small cell lung cancer pathway, which confirmed our model's validity. Besides, the substrate proteins were found to participate in some intriguing pathways, such as EGFR tyrosine kinase inhibitor resistance, Fc epsilon RI signaling pathway, platinum drug resistance, human T-cell leukemia virus 1 infection, PD-L1 expression and PD-1 checkpoint pathway in cancer, and Fc gamma R-mediated phagocytosis (Figure 6C). Enrichment for the above pathways predicts that our five signature genes may regulate immune infiltration. Since protein ubiquitination has been previously reported to influence innate and adaptive immunity [21], we further performed a detailed analysis of this process.

### Identification of immune infiltration in the low-risk group and high-risk groups

**Figure 7:**
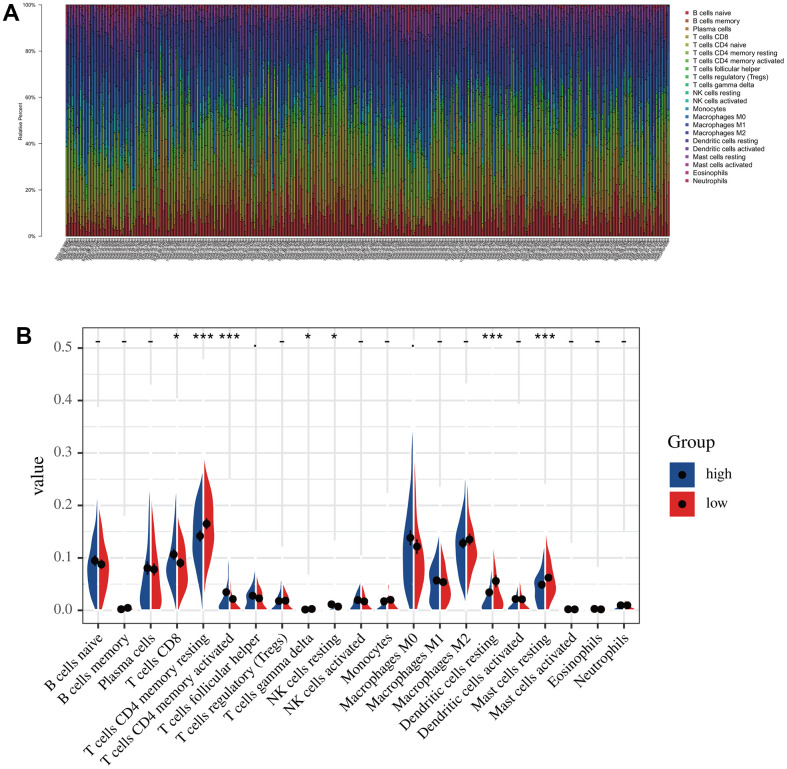
**The immune infiltration analysis in low-risk and high-risk groups.** (**A**) The distribution of the immune cells. (**B**) Differential analysis of immune cell infiltration in the high-risk and low-risk group.

According to the risk score, we divided all patients into high- and low-risk groups and then used the R package “CIBERSORT” to analyze infiltrated immune cells in each sample (Figure 7A). As shown in Figure 7B, the infiltration of CD8 cells was higher in the low-risk group than in the high-risk group, while NK cells, dendritic cells, and mast cells had higher infiltration levels in the high-risk group than in the low-risk group. However, the activation levels of these cells were not significantly different between the two groups group. In contrast, CD4 memory cells were significantly more activated in the low-risk group than in the high-risk group (Figure 7B). The above differences in these immune cells may be associated with differences in overall survival and tumor resistance to multiple treatments.

## DISCUSSION

Protein ubiquitination has been reported to be involved in various biological processes to regulate the growth or death of tumor cells [22]. E3s/DUB is involved in the regulation of numerous pathways by ubiquitinating substrates or changing the level of ubiquitination. Deepening further research and understanding of such E3s/DUB will help to expand existing cancer therapeutic targets and effective biomarkers, so it is worth exploring a prognostic prediction model for patients based on E3s/DUB. Although some signature gene-based prediction models for lung adenocarcinoma were recently reported [23–25], no study has focused on protein ubiquitination in LUAD. In the present study, we constructed an E3s/DUB-related signature gene model to predict prognosis and further analyzed the functional impact.

We systematically investigated the expression levels of E3s/DUB-related genes in lung adenocarcinoma patients and their relationship with overall survival. We constructed a brand-new five signature gene (WDR86, TRIM2, TRIM17, RNF144B, and UHRF1)-based prediction model for the training group and then validated the model in the test group. In this model, WDR86, TRIM2, and RNF144B were protective factors for risk score, while UHRF1 and TRIM17 were risk factors for LUAD. Among these genes, UHRF1, which has been demonstrated experimentally can promote disease progression and is associated with poor prognosis by affecting the cell cycle pathway in lung adenocarcinoma [26], which is consistent with our findings. However, previous studies have reported that TRIM2 mediates proliferation and metastasis of lung adenocarcinoma, by deubiquitinating and stabilizing Snail1 protein [27]. That is contradictory to our study in which TRIM2 was a risk protective factor. We suggest that TRIM2 binding to Snail1 may not be unique. Because deubiquitinase recognizes protein possessing the same specific domain. By affecting the ubiquitination levels of different substrate proteins, TRIM2 may play inconsistent cancer-promoting or cancer-inhibiting roles. When considering the impact of TRIM2 on patient survival, the combined effects of TRIM2 needs to be considered. More experiments are needed to investigate the combined effects of TRIM2 on survival in patients with lung adenocarcinoma. Besides, the specific role of other genes in the prediction model in lung cancer has not been reported, and further experiments on them can also help us to expand the understanding of the mechanisms of lung cancer development and progression. In our study, both Kaplan-Meier survival curves and ROC curves confirmed the reliability of our model in predicting survival. Then, we predicted the substrate proteins of the five signature genes to explore their functional impact. Interestingly, GO and KEGG analysis showed that the enriched pathways are associated with treatment resistance and the immune response and thus affect prognosis. Then, we studied immune cell infiltration in samples grouped according to risk scores calculated by our prediction model. Indeed, we observed some differences in immune cell infiltration scores, with the most significant difference being related to the activation of CD4 memory T cells, which was significantly increased in the low-risk group. This difference may have contributed to the better survival of patients in the low-risk group than those in the high-risk group. This finding is also consistent with previous reports showing that quiescent CD4 memory T cells exist in the microenvironment with tumors, where they can be activated by locally continuously released IL-12, allowing them to proliferate and secrete IFN-γ, leading to tumor cell death [28]. Of course, this study has some limitations, such as insufficient sample size and lack of experimental validation, which is also the focus of our future research.

In conclusion, we constructed an E3s/DUB-related signature gene-based model for predicting prognosis that performed well in predicting LUAD patient survival. Additionally, we identified the infiltrated immune cells significantly associated with the prognostic signature genes. These findings may provide novel insights for monitoring LUAD prognosis and guide the development of cancer immunotherapy.

### Data availability statement

The public datasets can be downloaded from TCGA (https://portal.gdc.cancer.gov/) and the IUUCD database (http://iuucd.biocuckoo.org/). The data used to support the findings of this study are available from the corresponding author upon request.

## MATERIALS AND METHODS

### Data downloading and processing

The RNA-seq data (HTSeq-FPKM) and clinical datasets for tumors (n=497) and normal samples (n=54) were downloaded from The Cancer Genome Atlas (TCGA) website (https://cancergenome.nih.gov/). E3s/DUB-related genes (n=1016) were identified from the IUUCD database (http://iuucd.biocuckoo.org/).

### Identification of differentially expressed E3/DUB-related genes

We used the R package “limma” to analyze differentially expressed genes (DEGs) between the tumor (n=397) and normal samples (n=54). The screening criteria were a corrected p-value < 0.05 and |log2FC| > 1.5. Then, the set of DEGs and an E3/DUB-related gene set were intersected using the R package “VennDiagram” to identify the E3/DUB-related genes that are differentially expressed in LUAD patients. The expression data for E3s/DUBs-related DEGs in all samples were plotted by R package “ggplot2” and “pheatmap”.

### Patient grouping and establishment and validation of the prediction model

Mean gene expression levels for samples with the same patient ID were calculated by the R package “limma”. Repeated patient samples and samples with insufficient follow-up information were excluded (n = 43). Then, the package “caret” was used to randomly divide the tumor samples into the training group (n=227; 50%) and test group (n=227; 50%). We identified E3/DUB-related DEGs associated with overall survival by the univariate Cox regression method after combining gene expression and response to clinical treatment information (*P* < 0.1). Moreover, we performed LASSO-Cox regression using signatures derived from univariate Cox regression and established prognostic signature formulae to avoid overfitting. LASSO regression was performed by the R package “glmnet” to identify robustly expressed genes. The parameter for LASSO was selected by ten-fold cross-validation. Subsequently, multivariate Cox regression analyses (*P* < 0.05) of these robustly expressed genes were performed to construct the risk score model. The risk scores of patients in the training group were calculated, and the median value of the risk score was used to divide the patients into high-risk and low-risk groups. Risk scores for patients in the test group were calculated as described above, and the same cutoff score was used for grouping. Kaplan-Meier analysis with the log-rank test and a ROC curve was used to detect the reliability of the model. A heatmap showing signature gene expression in the two groups was generated with the R package “survival”.

### Prediction and functional enrichment analysis of substrate proteins

The substrate protein of E3s/DUB is predicted using “UbiBrowser” (http://ubibrowser.ncpsb.org). The substrate proteins with the top 20 prediction scores were then selected for functional enrichment analysis. Gene Ontology (GO) and Kyoto Encyclopedia of Genes and Genomes (KEGG) analyses were performed with the R package “clusterProfiler”.

### Analysis of immune infiltration between the low-risk and high-risk groups

To understand the relationship between the model and infiltrated immune cells, we characterized immune cell composition using the R package “CIBERSORT”. The low-risk and high-risk groups were divided according to risk scores, and differentially infiltrated immune cells were identified. Here, a P-value < 0.05 was considered to be statistically significant.

### Statistical analysis

All statistical analyses are performed with R version 4.0.2 and the attached packages. The p-value and hazard ratio (HR) for survival analysis were derived from Cox regression. LASSO (least absolute shrinkage and selection operator) regression was used to filter the robustly expressed genes. The student’s t-test was used to explore the differences between the two groups. Moreover, the log-rank test was used for Kaplan-Meier survival analysis.

## Supplementary Material

Supplementary Figure 1

Supplementary Table 1
