# Supplementary material for: A novel protein ubiquitination-related five-gene signature predicts overall survival in patients with lung adenocarcinoma
Source: Aging (Albany NY). 2021 Mar 10;13(6):8510–23. doi: 10.18632/aging.202663 (PMC8034934; doi:10.18632/aging.202663)
Supplement: Supplementary Figure 1 [file aging-13-202663-s001.pdf]

SUPPLEMENTARY FIGURES

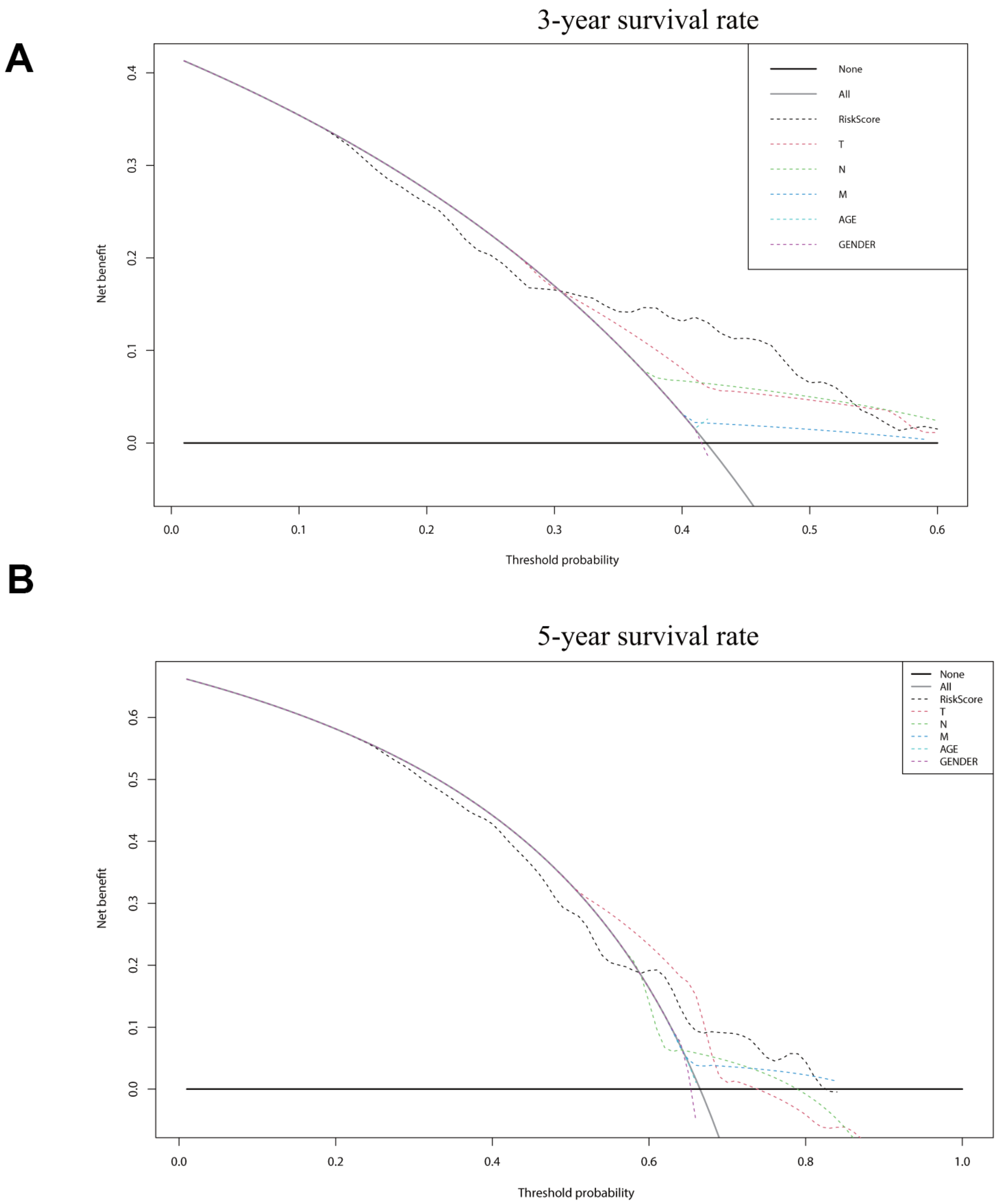

Supplementary Figure 1. Decision curve analysis of risk scores and other clinical factors. (**A**) Decision curve analysis of 3-year survival. (**B**) Decision curve analysis of 5-year survival.
